# Management and outcomes of bronchiolitis in Italy and Latin America: a multi-center, prospective, observational study

**DOI:** 10.1007/s00431-024-05530-6

**Published:** 2024-03-30

**Authors:** Anna Camporesi, Adriana Yock-Corrales, Jessica Gomez-Vargas, Damian Roland, Magali Gonzalez, Sandra Barreiro, Rosa Morello, Martin Brizuela, Danilo Buonsenso

**Affiliations:** 1 Division of Pediatric Anesthesia and Intensive Care, Buzzi Children’s Hospital, Milano, Italy; 2 Hospital Nacional de Niños “Dr. Carlos Saenz Herrera”, San José, Costa Rica; 3 SAPPHIRE Group, Department Population Health Sciences, Leicester University, Leicester, UK; 4 Paediatric Emergency Medicine Leicester Academic (PEMLA) Group, Children’s Emergency Department, Leicester Royal Infirmary, Leicester, UK; 5 Department Pediatrics Unit, Velez Sarsfield General Hospital, Buenos Aires, Argentina; 6 Department of Woman and Child Health and Public Health, Fondazione Policlinico Universitario A. Gemelli IRCCS, Largo A. Gemelli 8, 00168 Rome, Italy; 7 Center for Global Health Research Studies, Università Cattolica del Sacro Cuore, Rome, Italy

**Keywords:** Bronchiolitis, Europe, Latin America, RSV

## Abstract

We aimed to describe differences in the epidemiology, management, and outcomes existing between centers located in countries which differ by geographical location and economic status during to post-pandemic bronchiolitis seasons.  This was a prospective observational cohort study performed in two academic centers in Latin America (LA) and three in Italy. All consecutive children with a clinical diagnosis of bronchiolitis were included, following the same data collection form.  Nine hundred forty-three patients have been enrolled: 275 from the two Latin American Centers (San Jose, 215; Buenos Aires, 60), and 668 from Italy (Rome, 178; Milano, 163; Bologna, 251; Catania, 76). Children in LA had more frequently comorbidities, and only rarely received palivizumab. A higher number of patients in LA had been hospitalized in a ward (64% versus 23.9%, *p* < 0.001) or in a PICU (16% versus 6.2%, *p* < 0.001), and children in LA required overall more often respiratory support, from low flow oxygen to invasive mechanical ventilation, except for CPAP which was more used in Italy. There was no significant difference in prescription rates for antibiotics, but a significantly higher number of patients treated with systemic steroids in Italy.

*Conclusions*: We found significant differences in the care for children with bronchiolitis in Italy and LA. Reasons behind such differences are unclear and would require further investigations to optimize and homogenize practice all over the world.

**Table:** 

**What is Known:** *• Bronchiolitis is among the commest cause of morbidity and mortality in infants all over the world.*	
**What is New:** *• There are significant differences on how clinicians care for bronchiolitis in different centers and continents. Differences in care can be principally due to different local practices than differences in patients severity/presentations.* *• Understanding these differences should be a priority to optime and standardize bronchiolitis care globally.*

**What is Known:**

*• Bronchiolitis is among the commest cause of morbidity and mortality in infants all over the world.*

**What is New:**

*• There are significant differences on how clinicians care for bronchiolitis in different centers and continents. Differences in care can be principally due to different local practices than differences in patients severity/presentations.*

*• Understanding these differences should be a priority to optime and standardize bronchiolitis care globally.*

## Introduction

Seasonal epidemics of bronchiolitis respiratory syncytial virus (RSV) are the cause of substantial morbidity and mortality among children under 24 months old [1]. During the pandemic caused by SARS-CoV-2 in 2020/2021, the incidence of bronchiolitis in children decreased dramatically compared to pre-pandemic years globally [2–4]. Reports from Europe, South Africa, and South America showed reductions in RSV cases and hospitalizations due to nonpharmacological interventions; this phenomenon is also seen with other viruses like influenza and human metapneumovirus [5–8]. 

Reports from different countries have showed an atypical circulation of RSV after the year 2021 among children who needed hospitalization with an increase in the severity of the disease and a more severe impact in children less than 5 years old having their first RSV infection [6–8]. This has been reported by some researchers as an immunity dept because of a lack of exposure to different viruses during the previous years, leading to a large susceptible population of children [9, 10]. 

A multi-center prospective study in Italy, of the two last post-pandemic RSV seasons, showed a temporal difference between the two seasons with most children known to be healthy, less than 6 months old, 60% of them RSV-positive, and with a higher risk of hospitalization and PICU admission. The researchers concluded that these seasons were in line with pre-pandemic expectations [11]. 

There is a lack of information regarding bronchiolitis season in the post-pandemic years in regions like Latin America (LA), and whether there are differences in management and outcomes in this region compared with Europe. In this prospective study, we aim to describe epidemiological, treatment differences and outcomes existing between centers located in countries which differ by geographical location and economic status in a post-pandemic bronchiolitis season. The same data collection methodologies between European and Latin American countries have not been utilized previously. Surveillance of this disease and a determination of its burden is needed to implement future preventive interventions across a range of different geographical locations, including maternal and anti-RSV monoclonal vaccination.

## Methods

This is a multi-center prospective observational cohort study. The protocol was adapted by an open-access BronchSTART protocol developed by the PERUKI (Pediatric Emergency Research in the UK and Ireland) network, aiming to monitor real-time new bronchiolitis cases in the UK and Ireland since July 2021 [12] and adopted by four Italian University Hospitals in three different geographic areas (Northern Italy: Milano and Bologna; Central Italy: Rome; and Southern Italy: Catania) and two Hospitals in Latin America (San Josè, Costa Rica, and Buenos Aires, Argentina). The data of the Italian centers have been partially published previously [11, 13]. Economic status was classified as follows: Italy, high income; Costa Rica and Argentina, upper-middle income [14]. The season of enrollment is the first one after the burst of Covid pandemic: September 2021–April 2022 for Italy and March 2022–December 2022 for Latin America.

All consecutive children assessed in the participating institutions’ emergency departments (EDs) and receiving a clinical diagnosis of bronchiolitis or of a first episode of acute viral wheeze were included in the study. Bronchiolitis was defined as the presence of cough, tachypnoea or chest recession, and wheeze or crackles on chest auscultation.

Data have been collected on an online dataset in anonymized form. Given the nature of the study and the peculiar period characterized by the high workload in hospital settings and limited resources available, along with the need of having real time prospectively collected clinical data, each center was allowed to establish its own guidelines on the viral testing and therapeutic management of children (since type of treatment was outside of the purpose of the study).

Data were collected at time of the first hospital evaluation of the patient. Patients were followed up to 7 days after this first ED evaluation; if hospitalized at the time of presentation, through the clinical chart of the patient; if discharged home on the first ED contact, the families were contacted telephonically and asked about the subsequent evolution of the patient, including potential new ED accesses with potential hospital admission.

Demographic data, clinical history, date of initial assessment, main vital signs, treatments administered, outcome after first ED visit (discharge, admission to Short Stay Unit / Inpatient Ward/ High-Dependency Unit/Pediatric Intensive Care Unit), need and type of respiratory support, etiologies of the bronchiolitis (through nasopharyngeal swabs, including SARS-CoV-2) when tested, were collected.

The study was approved by the ethic committees of each country (Italy, protocol code Prof 0009995/22, ID 4730; Costa Rica CEC-HNN-018-2021, Argentina 2476/MSGC/2019).

### Statistical methods

Categorical variables were described as frequencies and percentages, and continuous variables were expressed as mean ±standard deviation or median (interquartile range) as appropriate. We used Fisher’s exact test or Pearsons’s chi-squared test to analyze categorical variables and Student’s *t*-test or Wilcoxon rank-sum test for continuous variables as appropriate. Multilevel mixed logistic regression has been performed for the outcomes “Cpap ventilation” and “mechanical ventilation,” with randomly varying effects on the center. Demographic as well as microbiological data have been used as covariates for the logistic, which was performed with a step-down method. Goodness-of-fit of the final models has been proved with the Hosmer-Lemeshow test. Data have been analyzed with STATA BE v. 18.0 (StataCorp, LLC, USA) and JMP^®^ 16.0.0.

## Results

During the considered season, 943 patients have been enrolled in the study among all centers: 275 from the two Latin American Centers (San Jose, 215; Buenos Aires, 60) and 668 from Italy (Rome, 178; Milano, 163; Bologna, 251; Catania, 76).

Children from Latin America were more often males and presented more often with prematurity and bronchopulmonary dysplasia, but not other comorbidities (Table 1). Two hundred sixty-nine patients had received palivizumab prophylaxis: 3 from Latin America and 266 from Italy.

**Table 1:** Baseline characteristics of the two cohorts. Data are presented as median (IQR) for continuous measures, and *n* (%) for categorical measures

	**Total**	**Latin America**	**Italy**	***p*-value**
	*N* = 943	*N* = 275	*N* = 668	
Age (months)	4 [2–9]	5 [2–10]	4 [2–9]	0.11
Male sex	505 (53.6%)	169 (61.5%)	336 (50.3%)	0.002
Prematurity	89 (9.4%)	39 (14.2%)	50 (7.5%)	0.002
Bronchopulmonary dysplasia	17 (1.8%)	10 (3.6%)	7 (1.0%)	0.012
Congenital cardiopathies	29 (3.1%)	13 (4.7%)	16 (2.4%)	0.065
Neuromuscular disorders	12 (1.3%)	6 (2.2%)	6 (0.9%)	0.11
Other comorbidities	78 (8.3%)	29 (10.5%)	49 (7.3%)	0.12

### Clinical presentation

Oxygen saturations (SpO2) upon presentation is available for 931 patients overall; of these, 854 had their first measurement in room air and 77 in oxygen. Median SpO2 in those in air was significantly lower in children from LA (94 (90–97) % vs. 96 (94–98) %; *p* < 0.001). Seventy-seven patients had their first Spo2 recording in oxygen therapy (63 from LA and 14 from Italy). Their Spo2 levels were not significantly different.

One hundred eighty-seven patients received a blood gas analysis in the ED; mean arterial pH was 7.37 ± 0.5 in Italy and 7.30 ± 0.11 in LA (*p* = 0.003); mean venous pH was 7.32 ± 0.11 in Italy and 7.31 ± 0.09 in LA (*p* = 0.43). Mean arterial pCO2 was 43.57 ± 12.01 mmHg in Italy vs. 58 ± 15.5 in LA (*p* = 0.10). Mean venous pCO2 was 47.49 ± 14.07 mmHg in Italy versus 53.88 ± 17.76 in LA (*p* = 0.21).

A virus test was performed in the ED in 545 patients, 377 in Italy and 168 in LA; respiratory syncytial virus was present in 335 (254 in Italy and 81 in LA).

### Respiratory and nutritional support in the ED

Patients in the LA cohort globally required a higher level of support compared to the Italian ones, except for suction of the upper airway which was more often performed in Italy. Support required during ED stay is described in Table 2.

**Table 2:** Support required in the two cohorts

**Support in the ED**	**Total**	**Latin America**	**Italy**	***p*-value**
	*N* = 943	*N* = 275	*N* = 668	
Suction	142 (15.1%)	21 (7.6%)	121 (18.2%)	< 0.001
Feed/fluids	241 (25.6%)	94 (34.2%)	147 (22.0%)	< 0.001
*NG fluids*	4 (0.4%)	3 (1.1%)	1 (0.2%)	0.045
*IV fluids*	192 (20.4%)	110 (40.0%)	82 (12.3%)	< 0.001
Respiratory support	319 (33.8%)	166 (60.4%)	153 (22.9%)	< 0.001
*Oxygen (low flow)*	216 (23.0%)	119 (43.3%)	97 (14.6%)	< 0.001
*Oxygen (high flow)*	81 (8.6%)	39 (14.2%)	42 (6.3%)	< 0.001
*CPAP*	9 (1.0%)	2 (0.7%)	7 (1.1%)	0.64
*IMV*	35 (3.7%)	34 (12.4%)	1 (0.2%)	< 0.001

Data are presented as *n *(%)

*NG* nasogastric, *IV* intravenous, *CPAP* continuous positive airway pressure, *IMV* invasive mechanical ventilation

### Destination after ED visit

After the ED visit, a similar number of patients were discharged home in both cohorts (Italy, 41.4%; LA, 35.3%; *p* = 0.081), and a similar number of patients were admitted to a pediatric ward (Italy, 40.9%; LA, 45.5%; *p* = 0.19). A higher number of patients in LA were admitted to a PICU (12% compared to 3.1% in Italy; *p* < 0.001). No deaths in the ED were observed in any of the two cohorts.

### One-week outcomes

**Fig. 1:**
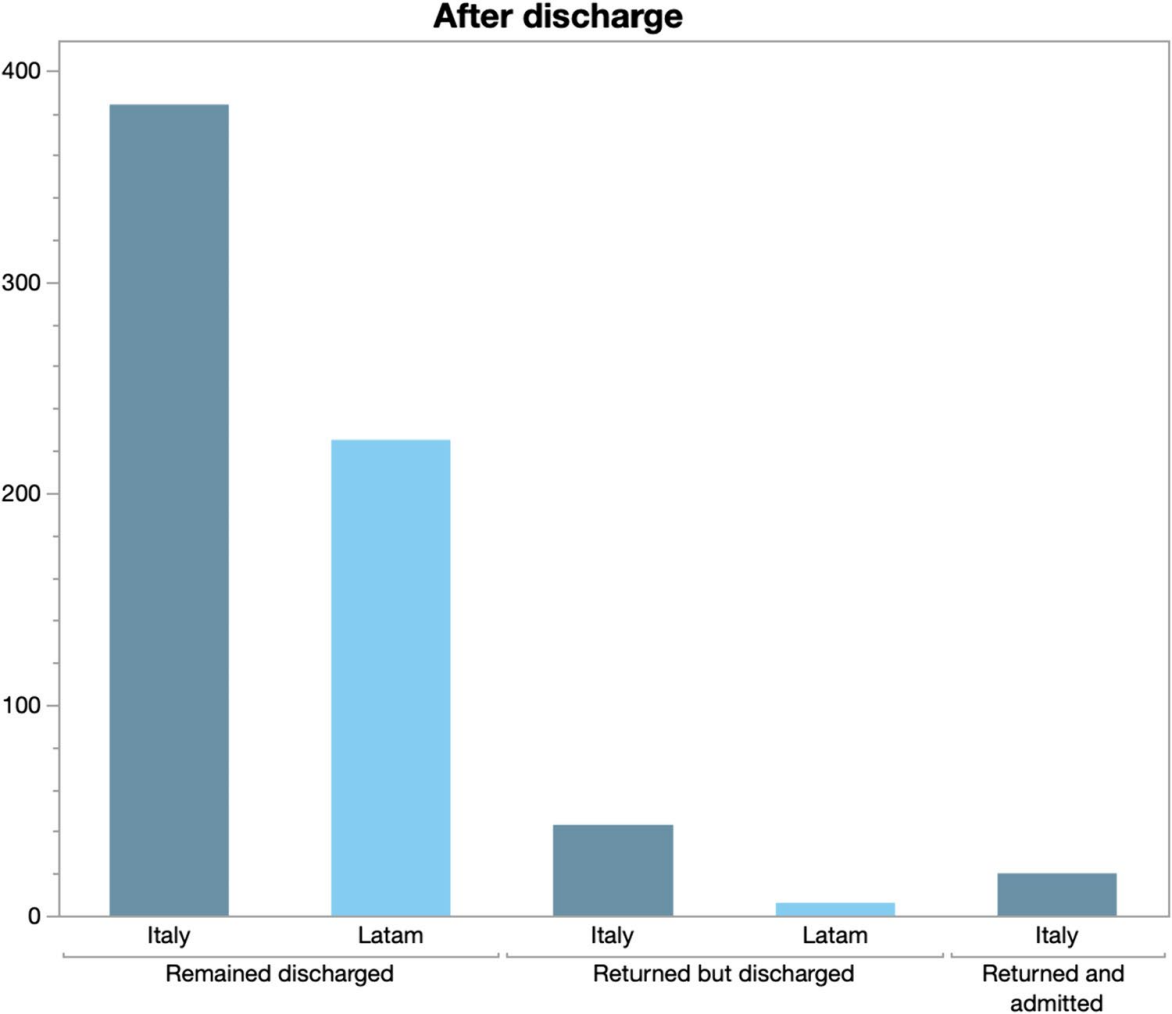
Number of re-assessments at 1 week discharge

Seven days after the first ED contact, of all the patients who had been discharged home during the first visit, 225 patients in LA and 385 of the Italian patients remained discharged. Twenty-three patients in Italy and 4 in LA had returned to the ED and had been discharged again, while 9 patients in Italy had returned to ED and been admitted to the hospital (Fig. 1).

**Fig. 2:**
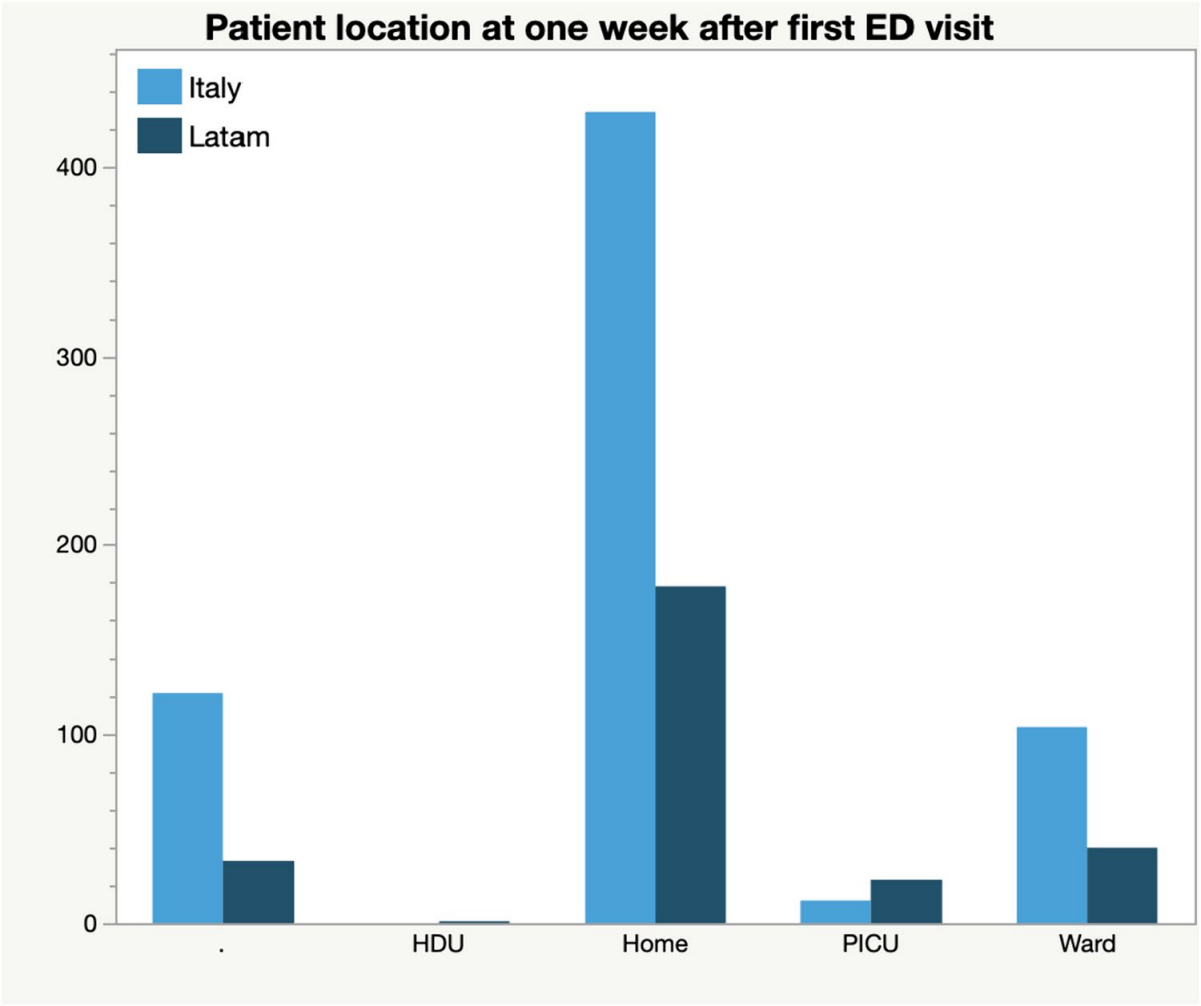
Patients location at 1 week time point after first assessment

Overall, at 7 days from ED visit, a significantly different number of patients in LA had been hospitalized in a ward (16.53% LA versus 19.05% in Italy, *p* < 0.001) or in a PICU (9.5% in LA versus 2.3% in Italy, *p* < 0.001) (Fig. 2).

### Global support

Table 3 shows the use of drugs and the level of support the two cohorts required overall. Patients in LA required overall more often respiratory support, from low flow oxygen to invasive mechanical ventilation, apart from CPAP which was more used in Italy. There was no significant difference in prescription rates for antibiotics, but a significantly higher number of patients treated with systemic steroids in Italy.

**Table 3:** Support needed in the cohort

**Global support**	**Total**	**LA**	**Italy**	***p*-value**
	*N* = 943	*N* = 275	*N* = 668	
Salbutamol	362 (38.4%)	89 (32.4%)	273 (40.9%)	0.015
Any steroid	188 (20.0%)	5 (1.8%)	183 (27.5%)	< 0.001
Antibiotics	205 (21.8%)	56 (20.4%)	149 (22.3%)	0.54
NG fluids	74 (8.3%)	10 (3.6%)	64 (10.4%)	< 0.001
IV fluids	272 (30.9%)	115 (41.8%)	157 (25.9%)	< 0.001
Oxygen, low flow	298 (31.7%)	137 (49.8%)	161 (24.2%)	< 0.001
Oxygen, high flow	198 (21.0%)	66 (24.0%)	132 (19.8%)	0.16
CPAP	50 (5.3%)	5 (1.8%)	45 (6.8%)	0.001
IMV	41 (4.4%)	38 (13.8%)	3 (0.5%)	< 0.001

*NG* nasogastric, *IV* intravenous, *CPAP* continuous positive airway pressure, *IMV* invasive mechanical ventilation

### Respiratory support

Overall, there was a significant difference between Latin America and Italy in the respiratory support needed.

The characteristics of the patients who required CPAP and invasive mechanical ventilation are reported in Table 4.

**Table 4:** Characteristics of patients supported with Cpap and mechanical ventilation, respectively

**Respiratory support**	**Total**	**No Cpap**	**Cpap**	***p*-value**	**No MV**	**MV**	***p*-value**
	*N* = 941	*N* = 891	*N* = 50		*N* = 900	*N* = 41	
Age (months)	4.0 (2.0–9.0)	4.0 (2.0–9.0)	3.0 (1.0–7.0)	0.012*	4.0 (2.0–9.0)	2.0 (1.0–5.0)	0.009*
Male sex	505 (53.7%)	478 (53.6%)	27 (54.0%)	1.00	478 (53.1%)	27 (65.9%)	0.15
Ex prematurity	89 (9.5%)	84 (9.4%)	5 (10.0%)	0.81	81 (9.0%)	8 (19.5%)	0.048*
BPD	17 (1.8%)	16 (1.8%)	1 (2.0%)	0.61	14 (1.6%)	3 (7.3%)	0.034*
Congenital cardiopathy	29 (3.1%)	27 (3.0%)	2 (4.0%)	0.66	26 (2.9%)	3 (7.3%)	0.13
Neuromuscular disorder	12 (1.3%)	11 (1.2%)	1 (2.0%)	0.48	9 (1.0%)	3 (7.3%)	0.013*
Other comorbidities	77 (8.2%)	73 (8.2%)	4 (8.0%)	1.00	74 (8.2%)	3 (7.3%)	1.00
Spo2	94.8 (6.7)	94.9 (6.8)	93.9 (6.2)	0.33	94.9 (6.8)	94.4 (4.1)	0.68
pH	7.33 (0.09)	7.33 (0.09)	7.32 (0.08)	0.47	7.34 (0.08)	7.31 (0.12)	0.058
CO_2_	47.5 (14.8)	47.9 (14.9)	44.9 (14.5)	0.50	45.7 (12.9)	54.6 (19.4)	0.018*
RSV	418 (44.4%)	378 (42.4%)	40 (80.0%)	< 0.001*	393 (43.7%)	25 (61.0%)	0.036*
Covid-19	52 (5.5%)	49 (5.5%)	3 (6.0%)	0.75	49 (5.4%)	3 (7.3%)	0.49
Adenovirus	8 (0.9%)	8 (0.9%)	0 (0.0%)	1.00	6 (0.7%)	2 (4.9%)	0.044*
Metapneumovirus	30 (3.2%)	29 (3.3%)	1 (2.0%)	1.00	30 (3.3%)	0 (0.0%)	0.64
Rhinovirus	119 (12.6%)	111 (12.5%)	8 (16.0%)	0.51	105 (11.7%)	14 (34.1%)	< 0.001*
Influenza virus	31 (3.3%)	30 (3.4%)	1 (2.0%)	1.00	30 (3.3%)	1 (2.4%)	1.00

Data are presented as mean (SD) or median (IQR) for continuous measures, and *n* (%) for categorical measures

*BPD* bronchopulmonary dysplasia, *RSV* respiratory syncitial virus

*Statistically significant

Multilevel mixed-effects logistic regression was conducted for the outcomes “CPAP ventilation” and “MV” (Mechanical ventilation). For CPAP, the only significative risk factor was presence of neuromuscular disease (OR, 58.91; 95% CI, 8.27–419.36; *p* < 0.001). Intraclass correlation (ICC) was 0.17. For MV, risk factors were prematurity (OR 4.47; 95% CI, 1.75–11.38; *p* = 0.002) and RSV presence (OR 6.62; 95% CI, 2.81–15.58; *p* < 0.001). ICC was 0.8. For the outcome “MV,” results are shown in Table 5.

**Table 5:** Results of the multivariable logistic regression for the outcome “Mechanical ventilation”

**MV**	**Odds ratio**	***P*** > *z*	**[95% conf. interval]**	
Age (months)	0.91	0.015	0.84–0.98	
Neuromuscular disorders	13.25	0.001	2.71–64.66	
RSV	3.04	0.001	1.54–6.03	
Covid-19	9.70	0.001	2.51–3.74	
Adenovirus	12.89	0.005	2.18–76.21	
Rhinovirus	4.10	0.000	1.92–8.74	
_cons	0.03	0.000	0.017–0.05	

**Fig. 3:**
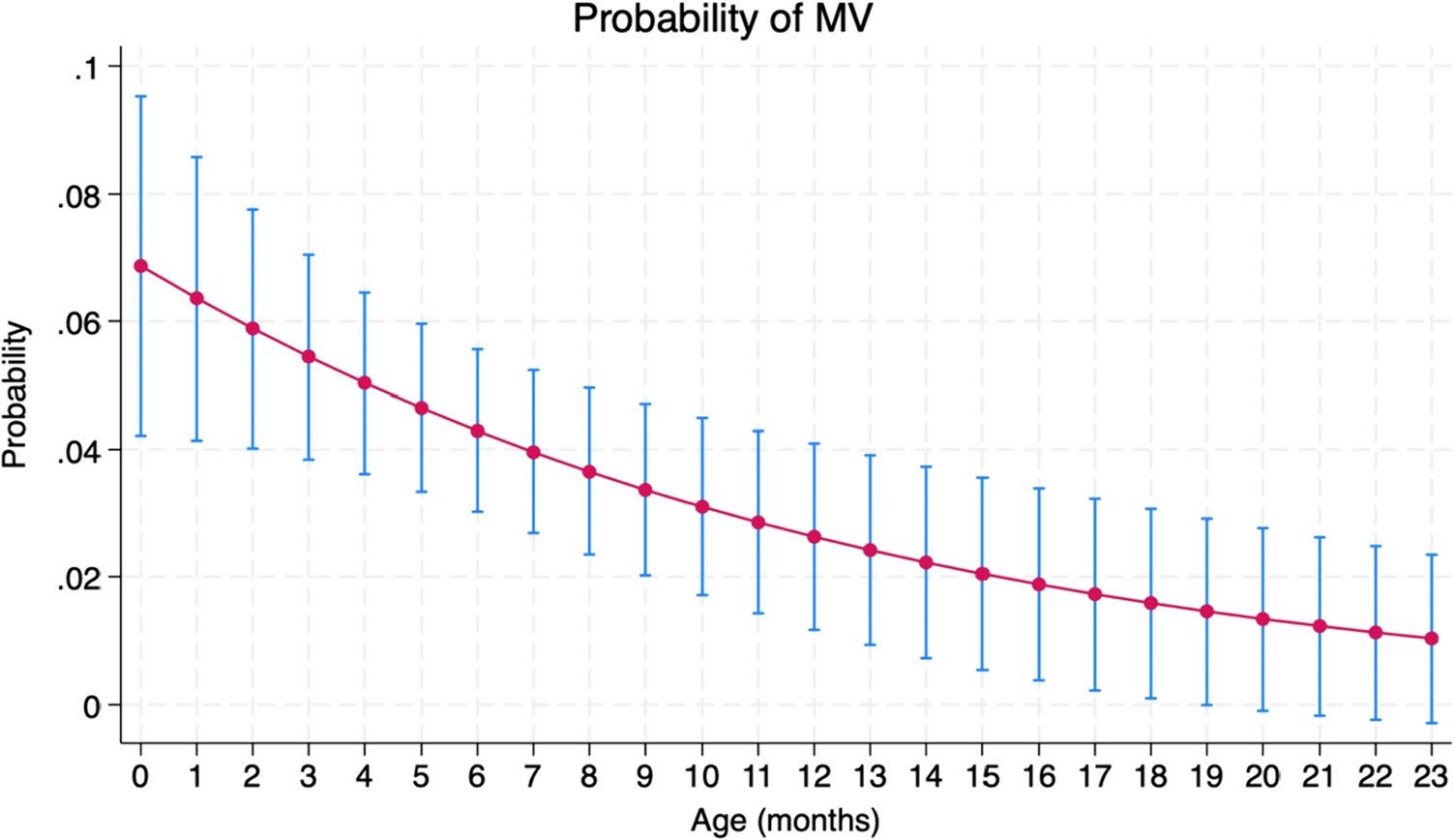
Probability of need for mechanical ventilation according to age of the patients enrolled

In multivariable analysis, age had a protective factor as expected on need for mechanical ventilation as shown in Fig. 3.

### Viral etiology

Overall viral etiology was similar only for the number of Covid infections but differed significantly for other respiratory viruses including RSV, which was significantly more diffuse in Italy (Table 6).

**Table 6:** Aetiologies distribution across centers

	**Total**	**Latam**	**Italy**	***p*-value**
	*N* = 943	*N* = 275	*N* = 668	
RSV	419 (44.4%)	86 (31.3%)	333 (49.9%)	< 0.001
SARS-CoV-2	52 (5.5%)	13 (4.7%)	39 (5.8%)	0.54
Adenovirus	8 (0.8%)	8 (2.9%)	0 (0.0%)	< 0.001
Human metapneumovirus	30 (3.2%)	18 (6.5%)	12 (1.8%)	< 0.001
Rhinovirus	120 (12.7%)	61 (22.2%)	59 (8.8%)	< 0.001
Influenza	31 (3.3%)	23 (8.4%)	8 (1.2%)	< 0.001

**Fig. 4:**
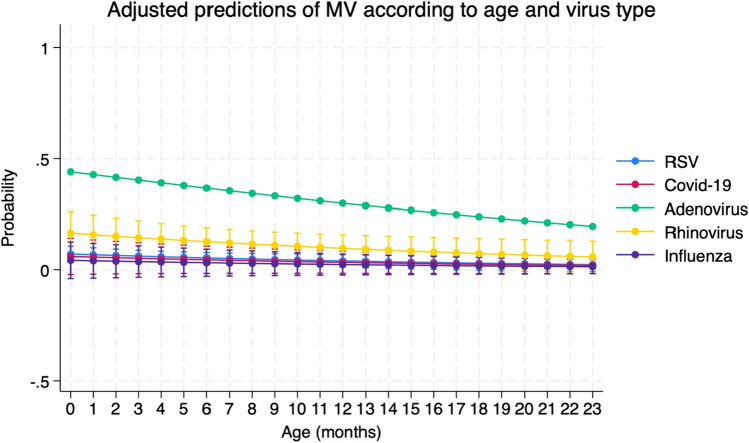
Prediction of need for mechanical ventilation according to age and type of virus

When considering the outcome “CPAP ventilation,” no virus had a significant odds ratio while when considering the outcome “invasive ventilation,” RSV, adenovirus, Covid-19, and rhinovirus had a significative odds ratio. A plot of interaction of age and type of virus in determining the prediction for invasive ventilation is shown in Fig. 4.

## Discussion

To our knowledge, this is the first study comparing cohorts of children from different continents (Europe and Latin America) assessed in hospital settings due to bronchiolitis, during the same season utilizing the same methodology. This study was possible because, after the release of a protocol for a prospective assessment of bronchiolitis in the UK [12], soon adopted also from Italian and Latin American centers that were collaborating on Covid-19 projects.

The most striking finding of our study is that, despite none of the children dying, the medical management of children with bronchiolitis in LA significantly differed compared to Italian centers. Although this study was not a trial investigating different interventions on clinical outcomes, the findings that LA children with bronchiolitis receive significantly higher use of mechanical ventilation, and lower use of CPAP and steroids, are interesting. In LA, in fact, 13.8% of children were intubated (vs. 0.5% in Italy < 0.001), and, as a consequence, PICU admissions were also higher (12% compared to 3.1% in Italy; *p* < 0.001). Conversely, Italian children received more frequently CPAP (6.8% vs. 1.8%, *p* 0.001) and steroids (27.5% vs. 1.8%, < 0.001). Differences in practice may be due to different historical care pathways in different countries, as well as different access to specific practices (e.g., less experience with or availability of CPAP and non-invasive ventilation in LA). Since the study was only focused on tracing local practices, epidemiology, and outcomes, we did not pre-specify criteria for the use of different interventions; therefore, we cannot conclude any causal relationships between the treatment used and outcomes. However, it is of interest that in Italy, hospitalized children more frequently receive steroids and CPAP and, as a possible consequence, need less frequent mechanical ventilation. Understanding such differences would be of great interest, as IV is associated with higher costs and potential barotrauma, need for sedation, longer stay in the PICU, and subsequent higher risk of hospital-acquired infections. Possible reasons for such a difference may be related to the lack of availability of resources for non-invasive ventilation (NIV), but also the higher need for human resources (and specifically of nurses specialized in managing PICU patients on NIV), which may be challenging in countries with less economic resources like LA. A recent review, in fact, although found scarce literature on the topic, still highlighted the pivotal role played by nurses in NIV-related decision-making and responsibilities throughout the ventilatory support process, from interface selection to weaning [15]. Therefore, our study opens the need for better investigating if in LA a less invasive approach is feasible and associated with similar outcomes but more long-term health and cost-related benefits. Of note, differences in respiratory support in patients with respiratory failure seen in different continents have been recently documented in adults [16].

To date, no clinical trials have been able to document significant benefits from one or more pharmacological treatments in children with bronchiolitis. As such, guidelines suggest only supportive management in these children and suggest avoiding inhalers, antibiotics, and steroids [17, 18]. Nevertheless, it is well known that these treatments are frequently overused in pediatric departments [19] and outpatient pediatric primary care centers [20] worldwide. Our study confirms that both in Italy and LA inhalers and antibiotics are frequently used in children hospitalized with bronchiolitis. However, an interesting difference between the two settings is that steroids are only rarely used in LA but frequently in Italy. The reasons behind these differences are difficult to explain and may be related to historical local practices. Also in this case, it is difficult to understand if the lower use of steroids can be associated with a higher need of PICU and IV in LA, as this is not a trial, but such differences are interesting and would deserve future investigation.

We also note in LA, while a significantly higher number of children hospitalized with bronchiolitis had comorbidities like prematurity, bronchopulmonary dysplasia, congenital abnormalities, and neuromuscular disorders, palivizumab was rarely used, compared with Italy (266 patients had received palivizumab prophylaxis in Italy and 3 in LA). This finding is surprising as, according to all guidelines, children with the mentioned comorbidities are eligible for palivizumab prophylaxis as at higher risk of severe bronchiolitis [21]. Such data may suggest that the major need of IV in LA is mostly due to a higher burden of high-risk children susceptible to RSV in LA due to the low use of preventive strategies in that setting, probably due to lack of resources or awareness [22]. This information would be extremely important since new and longer-lasting effective anti-RSV strategies, such as long-acting monoclonals and maternal vaccines, are expected to be available globally by the end of 2023 or beginning of 2024, which therefore may have a major positive impact particularly in LMICs [23, 24]. Nevertheless, it is important to highlight that not all bronchiolitis hospitalizations were associated with RSV, but other pathogens can lead to severe bronchiolitis [17, 18], as it was evident from the etiologies of bronchiolitis cases seen in our centers in a single season. As such, anti-RSV strategies will most probably have a big impact on bronchiolitis but will not eliminate entirely the burden of the disease on young children. Therefore, future studies are needed to find more effective treatments.

Our study has limitations to address. Although our study is based on a prospective observational approach, we could not investigate the effect of specific interventions, because we aimed to prospectively monitor cases according to local practices. Secondly, despite the multi-center design, our study may not reflect the management of bronchiolitis from a national perspective. However, the San Jose Hospital in Costa Rica is the only pediatric hospital in the country which admits all children that need hospitalization in the country, while the four Italian centers and the only Argentinian center only reflect regional care.

In conclusions, we found significant differences in the care for children with bronchiolitis in Italy and LA, with children in LA receiving more IV and PICU, and children in Italy more steroids and CPAP. Reasons behind such differences are unclear and would require further investigations to optimize and homogenize practice all over the world, to reduce health-related short- and long-term inequalities in children with bronchiolitis.

## Data Availability

Data are available upon request to the corresponding author.
